# Re-study of *Guangdedendron micrum* from the Late Devonian Xinhang forest

**DOI:** 10.1186/s12862-022-02021-w

**Published:** 2022-05-23

**Authors:** Xue Gao, Le Liu, Min Qin, Yi Zhou, Lei Mao, De-Ming Wang

**Affiliations:** 1 Key Laboratory of Orogenic Belts and Crustal Evolution, School of Earth and Space Sciences, Peking University, Beijing, 100871 China; 2 School of Geoscience and Surveying Engineering, China University of Mining and Technology (Beijing), Beijing, 100083 China; 3 Institute of Geology and Paleontology, Linyi University, Linyi, 276000 China; 4 Anhui Geological Museum, Hefei, 230031 China

**Keywords:** *Guangdedendron micrum*, *Lagenicula*, Tree lycopsids, Xinhang forest, Late Devonian

## Abstract

**Background:**

*Guangdedendron micrum* is the Late Devonian tree lycopsid that made up Xinhang fossil forest in Anhui, China, showing the earliest stigmarian rooting system. Based on new specimens of this lycopsid, the roots bearing rootlets, terminal parts of stems, vegetative leaves and monosporangiate strobili containing megaspores are researched in detail.

**Results:**

The roots with four robust rhizomorphs are largely expanded and approach the size of those of the Late Carboniferous giant tree lycopsids in swampy forests. The rootlets along rhizomorphic axis leave oval to circular scars after abscission. Narrow-fusiform leaf cushions display a leaf scar, vascular bundle and ligule pit. Cylindrical megasporangiate strobili are borne singly, in pairs, or occasionally once-dichotomized. Of each megasporophyll, the pedicel consists of a keel and possibly undeveloped alations, and the long-triangular lamina presents a heel. Megasporangium is sessile and contains multiple *Lagenicula* megaspores with distinct spines and a large gula.

**Conclusions:**

*G. micrum* displays large terminal monosporangiate strobili probably adapted to turbulent condition, and its megasporophylls together with multiple *Lagenicula*-type megaspores hint a possible primitive evolutionary status. These characteristics provide new insights into the evolution of fertile traits of early lycopsids.

**Supplementary Information:**

The online version contains supplementary material available at 10.1186/s12862-022-02021-w.

## Introduction

The evolutionary radiation of vascular plants in the Devonian is one of the major events in the history of life, leading to the establishment of the terrestrial ecosystems [1, 2]. During the Devonian, trees originated and evolved independently in three major taxa, including pseudosporochnaleans (a kind of fern-like plants or possible stem ferns), archaeopteridaleans and lycopsids [3–6]. The Late Devonian is an important period for lycopsids, during which they rapidly evolved or diversified crucial traits such as bipolar growth, heterospory and arborescence [7, 8]. Arborescent lycopsids then dominated Carboniferous swamp forests ecosystem [9] and occupied the majority of biomass that turned into coals [10, 11]. However, Devonian forests preserved in-situ are rarely known and mostly limited to Euramerica [1, 5, 6, 12, 13], and only the Late Devonian (Frasnian) Svalbard forest was reported before 2019 to consist of in-situ fossil lycopsids [1].

South China is regarded as a diversity hotspot of Late Devonian lycopsids as well as a potential research region for arborescent lycopsid evolution and Devonian lycopsid forests [5, 14]. Recently, Xinhang forest, a Famennian in-situ forest, was reported from Xinhang Town, Guangde City, Anhui Province of China [15], which primarily consists of a species of lycopsid, *Guangdedendron micrum,* that bears the earliest stigmarian rooting system. With the progress of clay excavation by local company, we keep on collecting plant specimens in Xinhang area. Based on new fossils, this article further studies the vegetative and reproductive characteristics of *G. micrum*, and provides information about its rhizomorphs, rootlets, vegetative stems bearing leaves, and monosporangiate strobili with megaspores.

Systematics

Class Lycopsida

Order Isoëtales *sensu lato* Meyen, 1987

Suborder Dichostrobiles DiMichele and Bateman, 1996

Genus: *Guangdedendron* Wang et al. 2019 emend

Emended generic diagnosis (with emended and additional diagnoses in brackets):

Small tree lycopsid, dioecious and monocarpic. *Stigmaria*-type rhizomorph with four axes, each of which equally divides at least once and bears helical rootlets nearly undivided. Stems dichotomous into a simple crown with terminal and pendulous megasporangiate strobili. Vegetative leaves linear with entire margin. [Leaf cushions and] leaf bases narrow-fusiform in shape, helically arranged in parastichies. [Leaf cushion or leaf base bearing ligule pit and vascular bundle scar. Leaf scars oval.] Megasporangiate strobili single, paired [or occasionally once-dichotomized], cylindrical with closely helically arranged megasporophylls. [Each megasporophyll comprised of a keeled pedicel and an upturned lamina with a downturned heel. Megasporangium long ellipsoidal, sessile and borne singly on adaxial surface of pedicel. Each megasporangium containing multiple *Lagenicula*-type] megaspores with trilete rays [and a gula].

Type species: *Guangdedendron micrum* Wang et al. 2019 emend.

Holotype: PKUB16001a (designated by Wang et al. 2019, housed in Department of Geology, Peking University, Beijing, China).

**Fig. 1:**
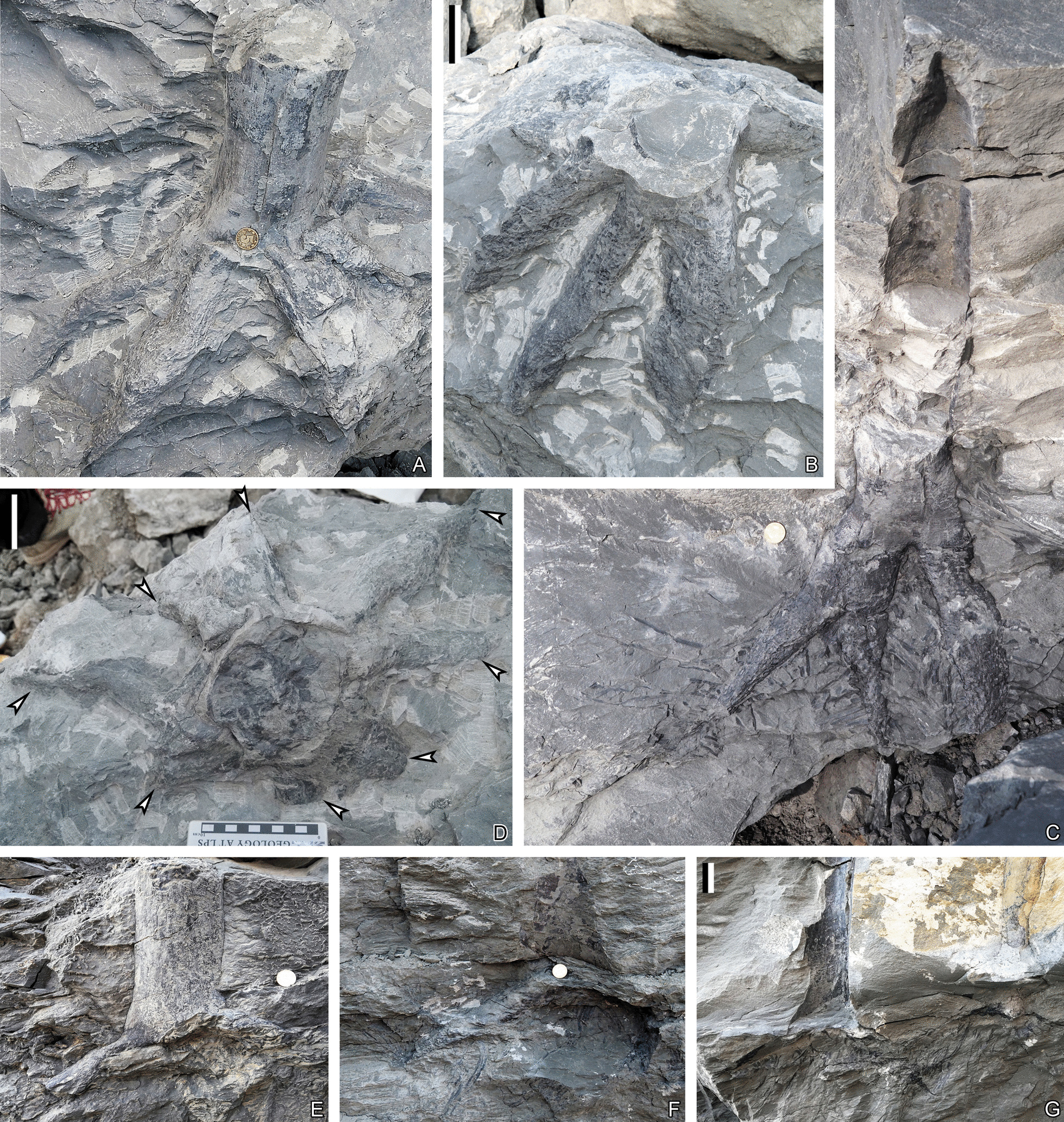
In-situ rooting systems of *Guangdedendron micrum* from Yongchuan mine. **A** Stem and connected rhizomorph axes. **B** Rooting system with branched rhizomorph axes. PKUB21015. **C** Stems with connected rhizomorph axes bearing rootlets. **D** Top view of the specimen shown in **A**, after removing the stem and surrounding rocks partially peeling off. A rooting system with four once-dichotomized rhizomorph axes. Arrows indicating 8 second-order branches. **E** Stem base and connected two rhizomorphic axes. **F**, **G** Rhizomorphic axes connected to moulds of stem bases and bearing rootlets. Scale bars: **B**, **D**, **G** (5 cm); diameter of the coin for scale: 2 cm (**A**, **C**, **E**, **F**)

**Fig. 2:**
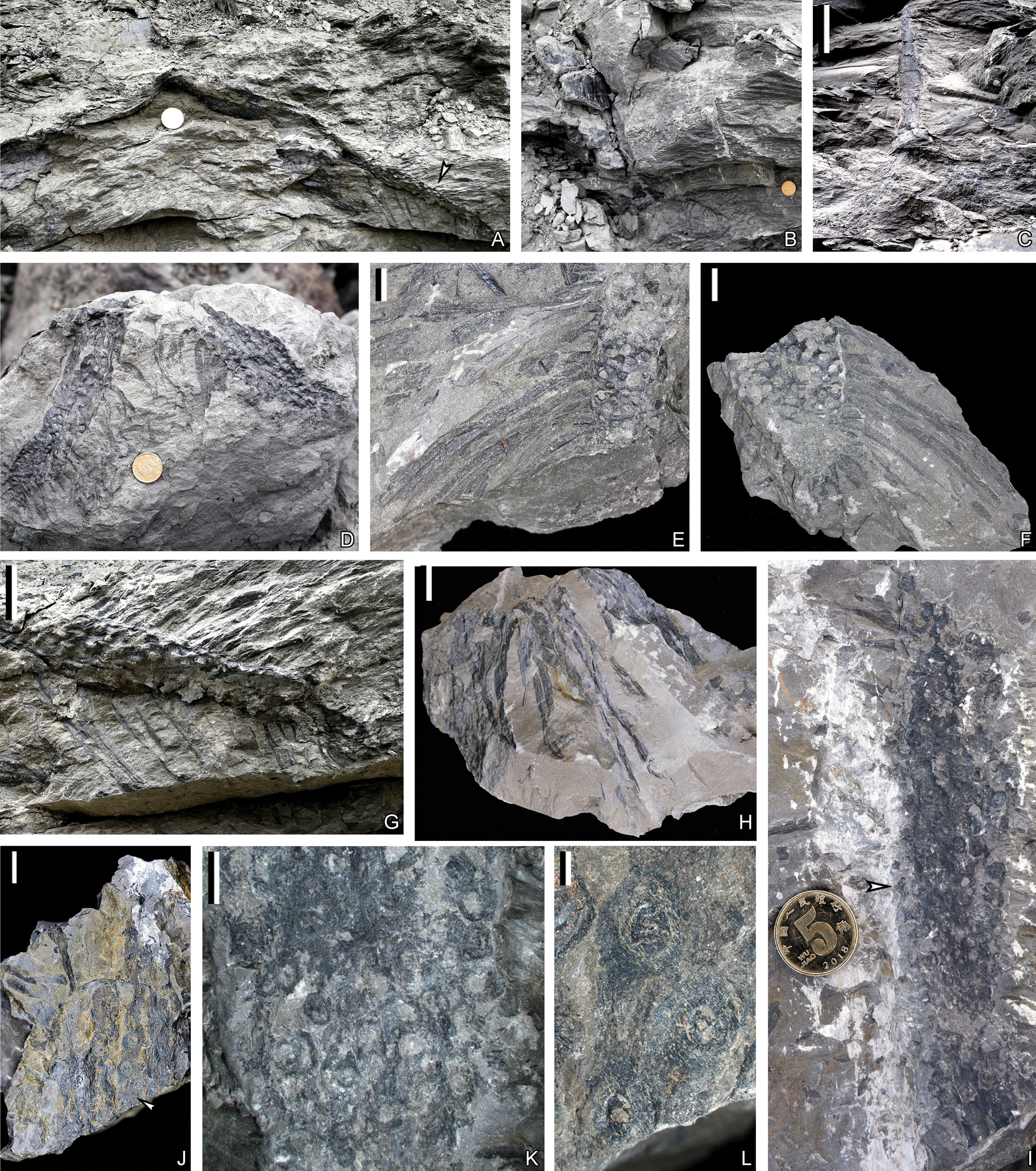
Rooting systems of *Guangdedendron micrum* from Yongchuan (**A**, **C**, **D**) and Jianchuan (**B**, **E**–**L**) mines. **A** An in-situ stem with two rhizomorph axes. Arrow indicating portion enlarged in (**G**). **B**, **C** In-situ stems with expanded bases and rooting systems. **D** Two rhizomorph axes with rootlets and rootlet scars. PKUB16151. **E**, **F** Part and counterpart of a rhizomorph axis with helical rootlets and circular rootlet scars. PKUB21016a, 21016b. **G** Enlargement of arrowed portion in (**A**), showing a rhizomorph axis with rootlets and helically arranged rootlet scars. **H** Part of the rooting system showing rootlets. PKUB21008. **I**, **J** Rhizomorphic axes bearing rootlets and circular rootlet scars in helix. Arrows in **I** and **J** indicating portions enlarged in **K** and **L**, respectively. PKUB21009, 21010. **K**, **L** Enlargement of arrowed portions in **I** and **J**, respectively. Circular rootlet scars in helix. Scale bars: **C** (10 cm), **E**–**G**, **J** (1 cm), **H** (2 cm), **K** (5 mm), **L** (2 mm); diameter of the coin for scale: 2 cm (**A**, **B**, **D**, **I**)

**Fig. 3:**
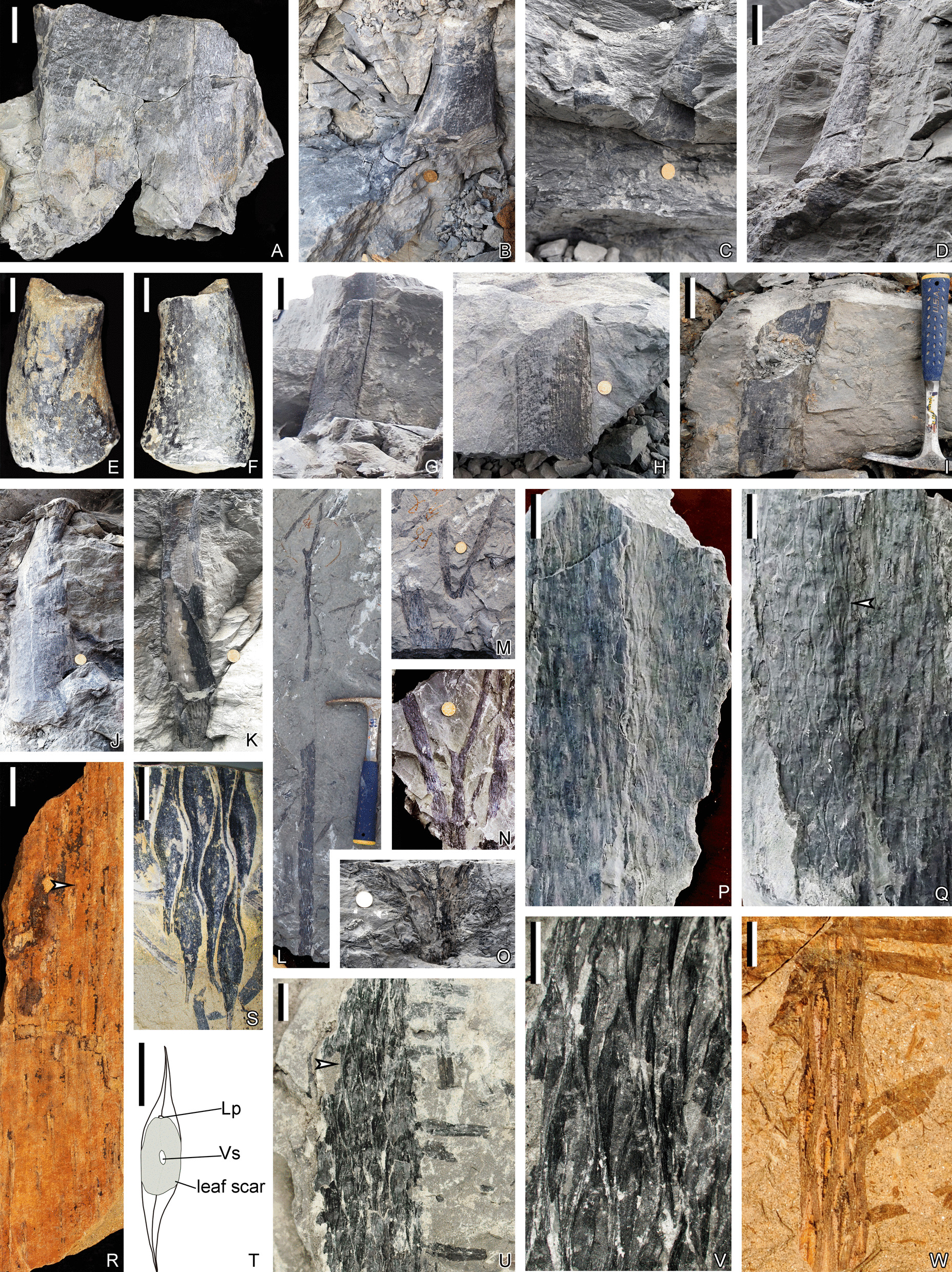
Stems and vegetative leaves of *Guangdedendron micrum*, from Yongchuan (**A**, **B**, **D**, **G**–**M**, **O**–**Q**, **S**, **U**, **V**) and Jianchuan (**C**, **E**, **F**, **N**, **R**, **W**) mines. **A**, **B** In-situ stems with expanded bases. PKUB21000, 21014. **C** Two adjacent in-situ stems with expanded bases. **D** An in-situ stem with an expanded base. **E**, **F** Two sides of a stem displaying basal expansion and helically arranged oval fissures of broken leaf cushions after leaf abscission. PKUB21004. **G**–**J** Stems perpendicular to the bedding plane. **H** YC-103. **K** An in-situ stem with leaf cushions. **L** A once-dichotomized stem. YC-101. **M**, **N** Twice-dichotomized stems. **M** YC-102. **O** A once-dichotomized leafy stem with leaf bases. **P**, **Q** Part and counterpart of helically arranged leaf cushions. YC-105, 104. **R** A stem with helically arranged leaf cushions. Arrow indicating portion enlarged in Additional file 1: Fig. S1I. PKUB16065. **S** Fusiform leaf cushions. PKUB21013. **T** Line illustration of a leaf cushion based on arrowed portion in **Q**. **U** Helically arranged leaf bases. Arrow indicating portion enlarged in **V**. PKUB21007. **V** Enlargement of arrowed portion in **U**, indicating fusiform leaf bases with middle vertical grooves in the lower part. **W** Helically arranged leaf bases, each showing a vertical groove in the middle. PKUB16052a. Scale bars: **A**, **E**, **F** (2 cm), **D**, **G** (5 cm), **P**–**R**, **U**, **W** (1 cm), **S**, **T**, **V** (5 mm); diameter of the coin for scale: 2 cm (**B**, **C**, **H**, **J**, **K**, **M**–**O**); length of the hammer for scale: **I** (28.6 cm), **L** (27.3 cm)

**Fig. 4:**
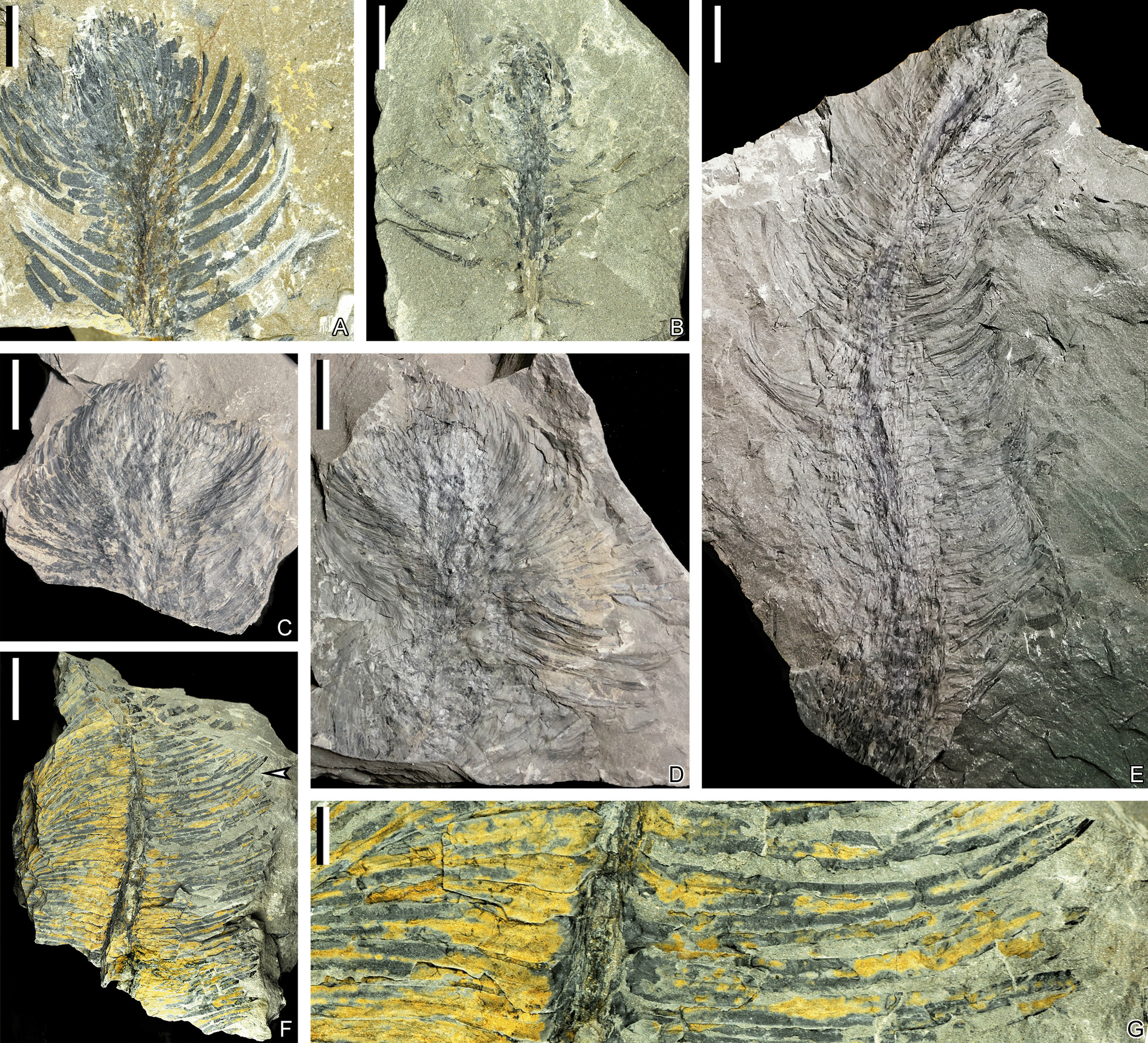
Vegetative leaves of *Guangdedendron micrum* from Jianchuan mine. **A**, **B** Terminal parts of vegetative axes bearing leaves. PKUB21001, 16067. **C**, **D** Part and counterpart of a terminal vegetative axis. PKUB21017a, 21017b. **E**, **F** Tapering vegetative axes with persistent linear leaves. Arrow indicating portion in **F** enlarged in **G**. PKUB21018, 16144. **G** Enlargement of arrowed portion in **F**, showing veins of vegetative leaves. Scale bars: **A**–**E** (2 cm), **F** (10 cm), **G** (1 cm)

**Fig. 5:**
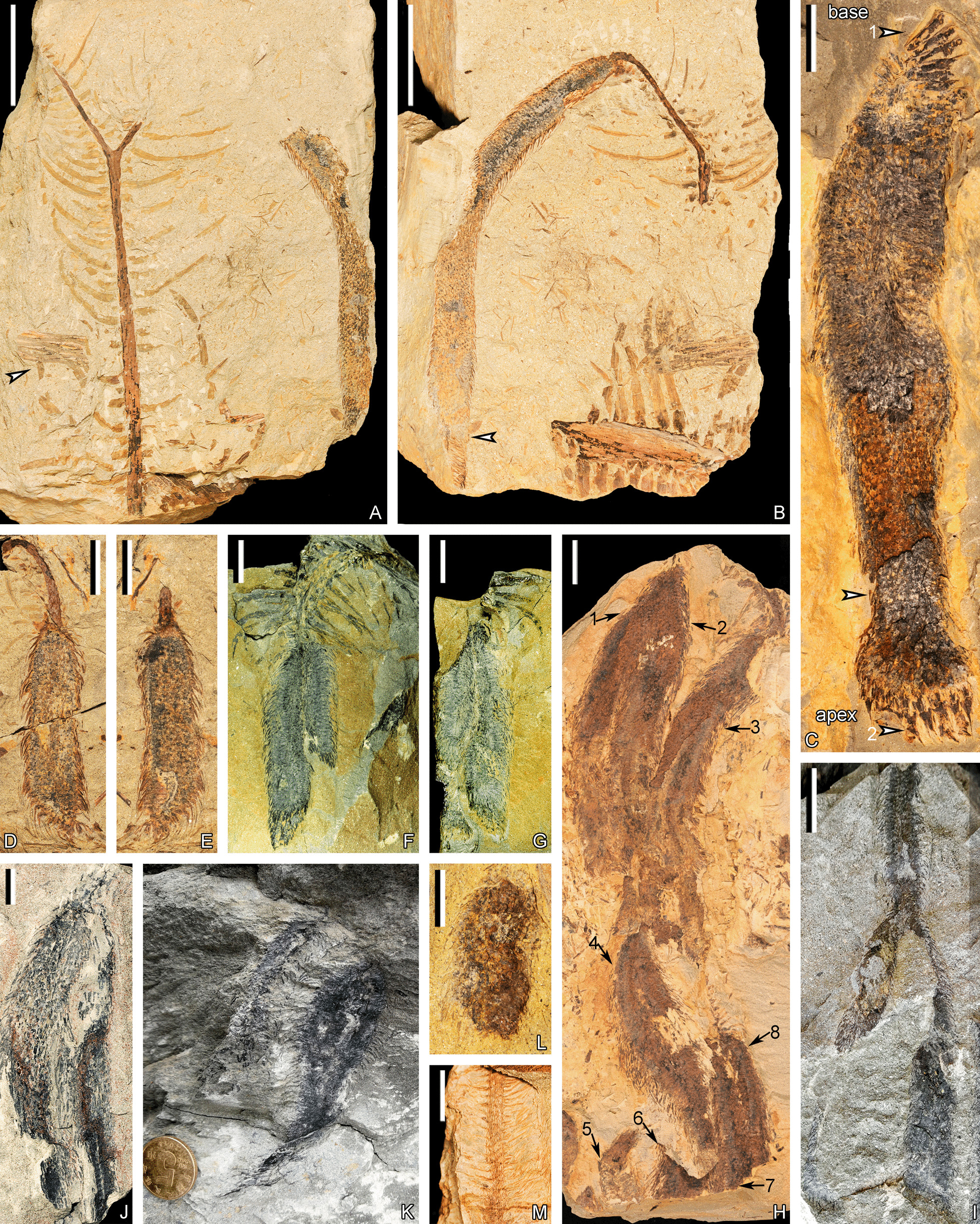
Fertile axes and strobili of *Guangdedendron micrum* from Jianchuan (**A**–**E**, **H**–**J**, **L**, **M**) and Yongchuan (**F**, **G**, **K**) mines. **A**, **B** Part and counterpart of a once-dichotomized axis bearing linear leaves and a single strobilus. The arrow in **A** indicating a leafy stem enlarged in Fig. 3W. Arrow in **B** indicating portion enlarged in Fig. 7D. PKUB16052a, 16052b. **C** A strobilus without basal fertile axis preserved. Arrows 1 and 2 indicating portions enlarged in Fig. 7E,  G, respectively. Middle arrow indicating portion where carbonaceous material was peeled for maceration and displayed in Fig. 8A. PKUB16053. **D**, **E** Part and counterpart of a strobilus terminating a fertile axis. PKUB16001a, 16001b. **F**, **G** Part and counterpart of terminal strobili in pairs, with sporophylls along central strobilar axis and persistent vegetative leaves on fertile axis. PKUB21002a, 21002b. **H** At least eight strobili (arrows 1–8) preserved in the same direction (1 and 2, 7 and 8 possible paired, respectively). PKUB16047. **I** A dichotomized strobilus. PKUB21011. **J** Terminal strobili in pairs. PKUB16035. **K** A single and a pair of strobili perpendicular to the layers. **L** A short strobilus may partially preserved. PKUB16065. **M** Strobilus displaying central strobilar axis. PKUB16020a. Scale bars: **A**, **B** (5 cm), **C**, **J**, **L**, **M** (1 cm), **D**–**I** (2 cm); diameter of the coin for scale: 2 cm (**K**)

Specimens examined here:

PKUB16001a, 16001b, 16020a, 16035, 16047, 16049, 16052a, 16052b, 16053, 16058, 16064, 16065, 16067, 16097, 16,099, 16141, 16144, 16151, 21000, 21001, 21002a, 21002b, 21004–21011, 21013–21015, 21016a, 21016b, 21017a, 21017b, 21018; YC101–105 (Figs. 1–5, 7, Additional file 1: Fig. S1).

Locality and horizon:

Jianchuan village, Xinhang Town, Guangde City, Anhui Province, China, Leigutai Member (Famennian) of Upper Devonian Wutong Formation.

Repository: Specimens numbered with PKUB and YC are separately housed at the Department of Geology, Peking University, Beijing, China and Anhui Geological Museum, Hefei, China, respectively.

Specific diagnosis (based on descriptions of Wang et al. [15] and this study): As for generic diagnosis. Rhizomorphs over 27.0 cm in depth. Rhizomorphic axes 8.3–31.0 cm long and 0.3–7.8 cm wide, forming angles of 19°–60° to ground surface. Rootlets up to 27.2 cm long and 7.0 mm wide. Rootlet scars circular and 2.5–3.9 mm in diameter. Preserved parts of in-situ stems reaching 88.0 cm in height and up to 18.7 cm in diameter, with rare dichotomy. Vegetative leaves 2.0–9.2 cm long and 1.2–9.0 mm wide, each with a single vein and an entire margin. The length–width ratios of leaf bases or leaf cushions ca. 6:1. Fertile axes with persistent leaves, terminated by strobili. Megasporangiate strobili cylindrical, with maximum length and width (excluding sporophyll laminae) of 23.4 cm and 3.0 cm, respectively. Strobilar axes up to 3.0 mm in width. Sporophyll laminae long-triangular in shape with the maximum length and width of 18.0 mm and 5.8 mm, showing entire margins. Basal portion of each lamina forming a downturned, inverted triangular heel. Sporophyll pedicels 6.0–8.0 mm in length, approximately perpendicular to strobilar axis. Pedicel displaying an abaxial keel 0.5–0.8 mm in height. Megasporangium horizontally elongated, 4.0–7.4 mm long. Megaspore ca. 1.5 mm in polar axis length, consisting of a smooth gula and a spherical body with spiny ornamentations.

## Results

### Description

**Fig. 6:**
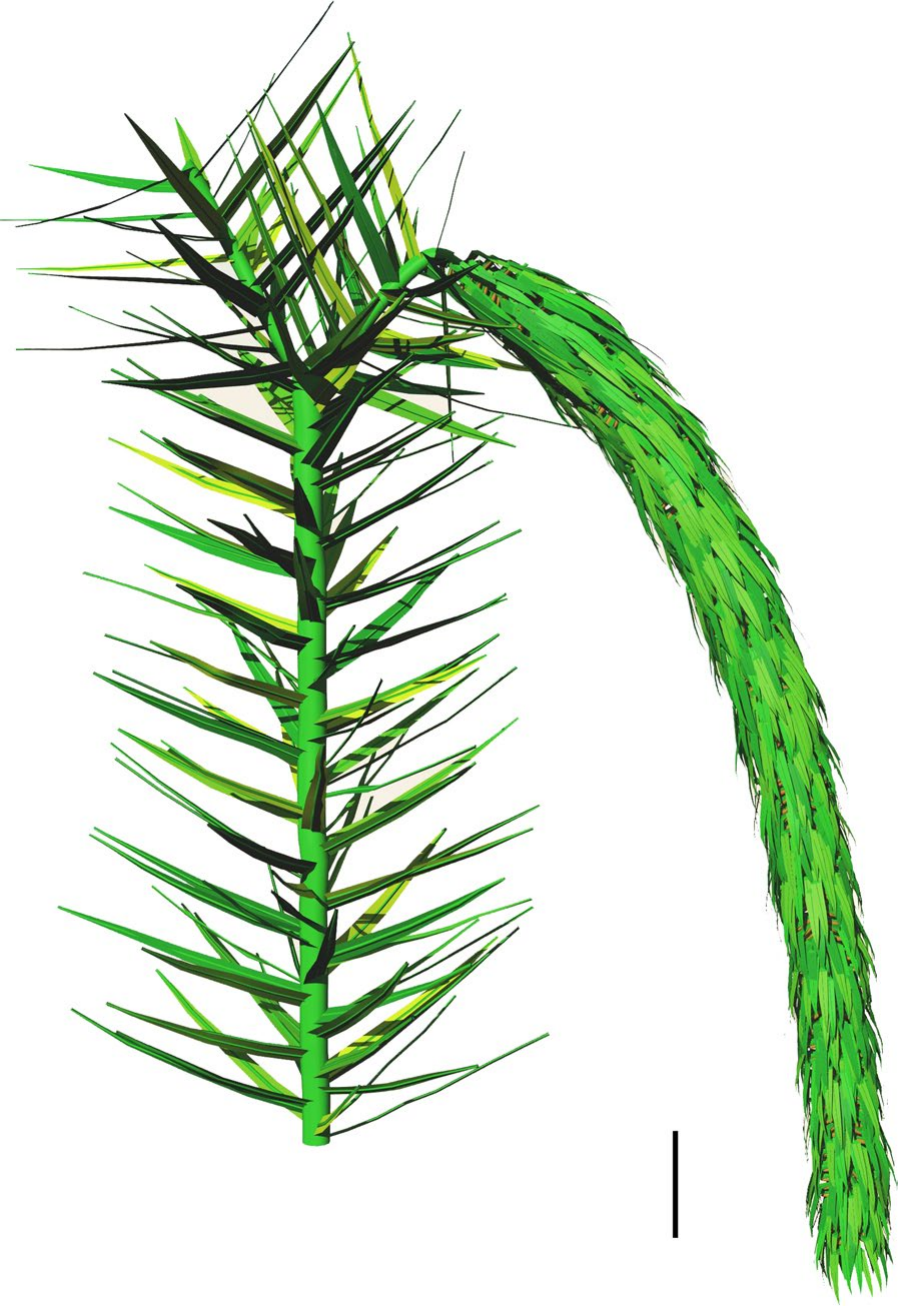
Reconstruction of the longest strobilus terminating the fertile axis after bifurcation, based on Fig. 5A, B. PKUB16052a, 16052b. Scale bar = 2 cm

**Fig. 7:**
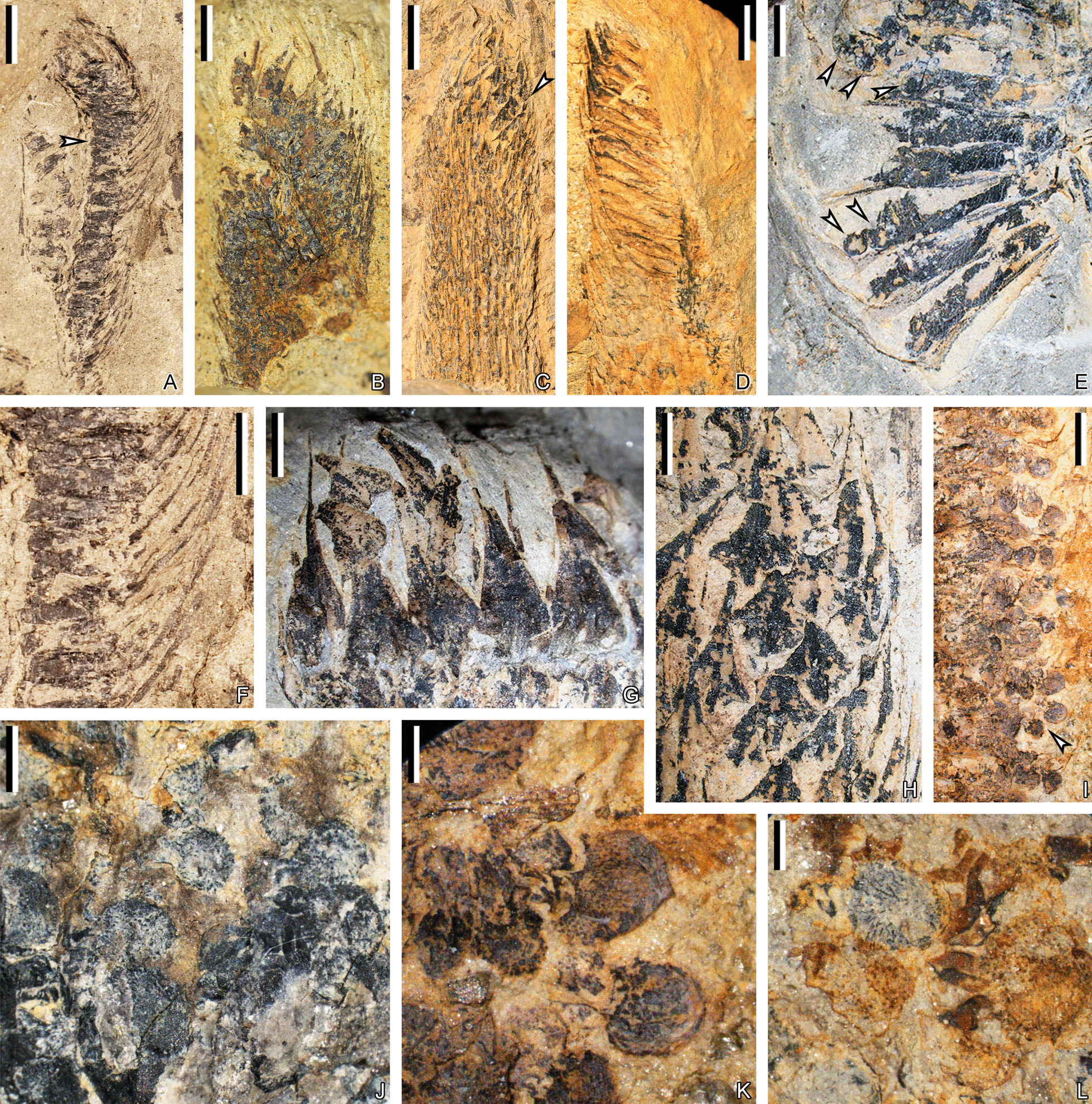
Sporophylls and spores of *Guangdedendron micrum* from Jianchuan mine. **A** A partially preserved strobilus, showing sporangia on the adaxial surface of sporophyll pedicels. PKUB16049. **B** A partially preserved strobilus, showing megaspores and sporophylls. PKUB16141. **C** Mid-upper part of a strobilus with helically arranged sporophylls in face and lateral view. Arrow indicating portion enlarged in **H**. PKUB16058. **D** Enlargement of portion in Fig. 5B (arrow, 180° rotated), showing sporophyll laminae and pedicel in lateral view. **E** Enlargement of portion in Fig. 5C (arrow 1, 180° rotated), respectively. Showing sporophyll laminae and pedicel in lateral view and partially preserved sporangia, arrows indicating megaspores. **F** Enlargement of arrowed portion in (**A**), showing sporangia. **G** Enlargement of portion in Fig. 5C (arrow 2, 180° rotated), showing sporophyll laminae in face view. **H** Enlargement of portion in (**C)** (arrow), showing sporophyll laminae in face view with downturned heels. **I** Part of a strobilus showing megaspores, the arrow indicating portion enlarged in (**K**). PKUB16020a. **J**, **K** Enlargement of arrowed portions in Additional file 1: Fig. S1Q and 7I, respectively. Showing megaspores and spiny ornamentations. **L** Megaspores with spiny ornamentations. Scale bars: **A**, **C**, **D** (1 cm), **B**, **F** (5 mm), **E**, **G**–**I** (2 mm), **J** (1 mm), **K**, **L** (500 μm)

**Fig. 8:**
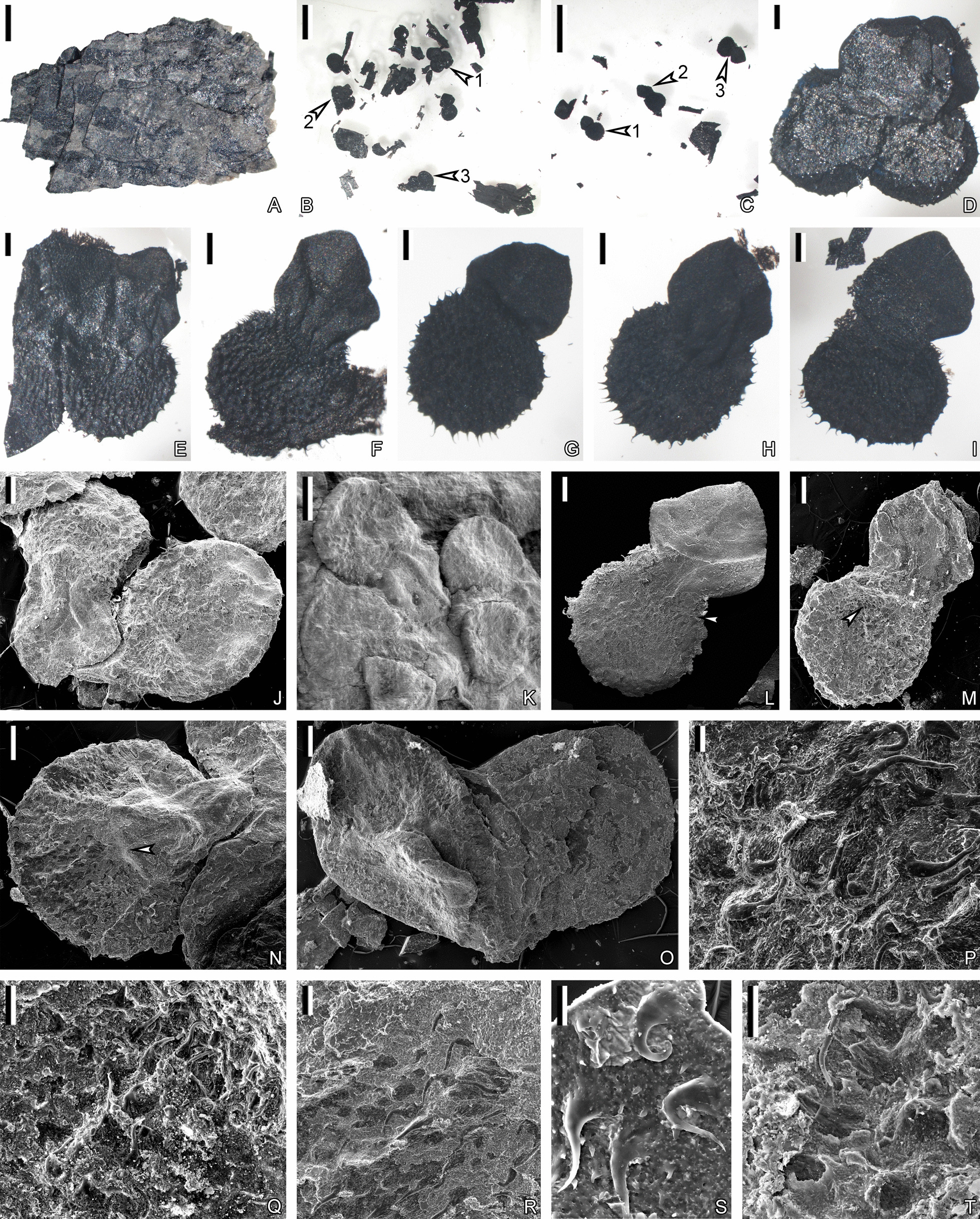
Megaspores of *Guangdedendron micrum* displaying ornamentations under LM after maceration (**B**–**I**) or under SEM (**J**–**T**), respectively. **A** A piece of carbonaceous fragment of strobilus peeled from Fig. 5C (middle arrow indicating portions, nearby the apex of strobilus) before maceration. **B**, **C** Megaspores after maceration of fragment in (**A**). Arrows in (**B**) and (**C**) indicating portions enlarged in (**D**–**I**), respectively. **D**, **E** Enlargement of portions in (**B**) (arrow 1 and 2), respectively, displaying megaspore clusters probably borne in tetrads. **F**–**I** Enlargement of portions in (**B**) (arrow 3) and **C** (arrow 1–3), respectively. Megaspore consisting of a body with spiny ornamentations and a prominent gula. **J**, **K** Megaspore clusters under SEM, probably borne in tetrads. **L**, **M** Megaspore in lateral view, each consisting of a body and a gula. Arrows in (**L**) and (**M**) indicating portions enlarged in (**P**) and (**Q**), respectively. **N**, **O** Megaspores in roughly proximal view. Arrow in (**N**) indicating portion enlarged in (**R**). **P**–**R** Enlargement of portions in (**L**) (arrow), **M** (arrow) and **N** (arrow), respectively. Showing spiny and tapered ornamentations. **S** Spiny and tapered ornamentations. **T** Enlargement of portion in Additional file 3: Fig. S3C (arrow 2), showing spiny ornamentations. Scale bars: **A** (1 mm), **B**, **C** (2 mm), **D**–**J**, **L**–**O** (200 μm), **K** (500 μm), **P**, **S** (20 μm), **Q**, **R**, **T** (50 μm)

The description in this article is based on a new fossil collection of *Guangdedendron micrum* as well as several specimens introduced in Wang et al. [15]. Plant organs described here include rooting system (Fig. 1, 2), stems and vegetative axes bearing leaves and leaf cushions or leaf bases (Figs. 3, 4), and strobili with the 3-D reconstruction (Figs. 5, 6) displaying megaspores (Figs. 7, 8).

The stigmarian rhizomorph has four evenly separated axes and then usually dichotomizes once (Fig. 1A–D). These rhizomorphic axes expand up to 41.1 cm in plane (Fig. 1D), over 27.0 cm in depth (Fig. 1C) and extend at 19°–51° to ground surface (Fig. 1C, E–G). Rhizomorph axes are up to 31.0 cm in length, and their first-order and second-order branches are 7.2–7.8 cm and 2.5–6.5 cm in diameter, respectively (Figs. 1A–D, 2A, C–G). Rootlets are helically arranged along rhizomorph axes, extend in different directions and taper gradually toward apices (Figs. 1C, F, G, 2A–H). Rootlets are over 12 cm in length, with a maximum width of 5.0 mm (Fig. 2A, C–H). When abscised, they leave oval to circular rootlet scars 2.5–3.9 mm in diameter on rhizomorphic axes (Fig. 2–I–L). Rootlets appear unbranched in most cases. No root hairs have been observed.

Stems are preserved as compressions or erect casts, up to 73.1 cm long and 1.1–12.2 cm in diameter except for the expanded bases (Fig. 3A–O; Additional file 1: Fig. S1A–F, H). The expanded bases are connected to rhizomorphs (Fig. 3B–D). Dichotomies are occasional, and at most continuously twice observed on stems and at angles of 13°–43° (Fig. 3L–O; Additional file 1: Fig. S1E, F). Stems or axes display leaf cushions (Fig. 3K) or leaf bases (Fig. 3O) when vegetative leaves have been abscised or not, respectively. Sometimes only oval or elongate fissures can be recognized along the stems due to poor preservation (Fig. 3E, F, Additional file 1: Fig. S1G, H). Leaf cushions are fusiform in shape, 15.6–22.7 mm long and 2.6–4.3 mm wide (Fig. 3P–S). Leaf scars are oval, 6.5–8.1 mm in length and 3.5–3.7 mm in width, appearing on the middle portion of leaf cushions (Fig. 3P, Q, T). The leaf scar shows a depressed ligule pit (Lp) on the top and a vascular bundle scar in the middle (Fig. 3P, Q, T; Additional file 1: Fig. S1I). Leaf bases are fusiform and somewhat narrower, 14.0–25.0 mm in length and 3.0–3.7 mm in width, with the ratio of length to width ca. 6:1 (Fig. 3U–W). Each leaf cushion or leaf base bears a longitudinal groove in the lower part (Fig. 3P, Q, V, W). Leaf cushions or leaf bases are helically arranged in parastichies, forming angles of 65°–80° with the horizontal line (Fig. 3P–S, U–W; Additional file 1: Fig. S1J).

Linear vegetative leaves along stems possess entire margins, 3.3–8.5 cm long and 2.9–9.0 mm wide, and depart at angles of 59°–98° from stems (Figs. 3U, W, 4E, F, 5A, B, D, F). Leaves of similar shape are densely arranged along slender terminal twigs (Fig. 4A–D), indicating terminal portion of a possible juvenile plant. Each leaf has an obvious single vein extending from base to apex (Fig. 4B, G).

Fertile axes terminated by strobili are up to 6.4 cm long and 2.1–5.5 mm wide (Fig. 5A, B, D–F). An axis, 17.6 cm long and 3.5–7.8 mm wide, produces two daughter axes with half the width of the parent and at angles of ca. 60° (Fig. 5A, B). The vegetative leaves depart at angles of 70°–85° from fertile axes and curve distally (Fig. 5A, B, D, F; Additional file 1: Fig. S1L, O).

Pendulous strobili are borne singly (Fig. 5A, B, D, E, H, K; Additional file 1: Fig. S1K, L, O), in pairs (Fig. 5F–H, J, K; Additional file 1: Fig. S1M, N) or occasionally once-dichotomized (Fig. 5I; Additional file 1: Fig. S1Q). Strobili are cylindrical and slightly curved, up to 23.4 cm in length and 0.9–2.4 cm in width (excluding sporophyll laminae) (Fig. 5A–L; Additional file 1: Fig. S1K–Q). The longest strobilus terminating the fertile axis (Fig. 5A, B) is reconstructed in Fig. 6. Sporophylls are helically and compactly arranged along strobilar axes that are 1.2–3.0 mm wide (Figs. 5M, 7A, D). Each sporophyll in lateral view shows a horizontal pedicel, from which a lamina arises at an angle of ca. 110° (Fig. 7D–F). The lamina is long-triangular in front view and tapers acropetally, 13.0–18.0 mm long and 2.4–5.8 mm at the widest part (Fig. 7G, H; Additional file 2: Fig. S2). The lamina forms a downturned heel at the base, which is 0.8–1.0 mm high and 2.3–2.5 mm wide (Fig. 7H). The pedicel is 6.0–8.0 mm in length (Fig. 7D–F), and shows an abaxial keel 0.5–0.8 mm high (Fig. 7D, E).

All the strobili in our collection only exhibit megaspores and are thus megasporangiate. Each megasporangium is sessile and long-ellipsoidal, 4.0–7.4 mm long and over 1.3–1.5 mm high (partially preserved or covered in height), and attached to the adaxial surface of sporophyll pedicel (Fig. 7E, F). The upper portions of megasporangia are usually incompleted because of overlap by the adjacent sporophyll(s). In-situ megaspores are sometimes observed within strobili, displaying circular shapes (Fig. 7I, J; Additional file 1: Fig. S1Q) and spiny ornamentations (Fig. 7J–L). A megasporangium contains multiple megaspores (Fig. 7B, E, arrows) considering their sizes.

*Lagenicula* megaspores are observed under LM after maceration (Fig. 8A–I) or under SEM (Fig. 8J–T; Additional file 3: Fig. S3), and are probably born in tetrads (Fig. 8D, J, K; Additional file 3: Fig. S3A). Each megaspore is composed of a spherical body and a prominent gula in lateral view (Fig. 8F–I, L, M; Additional file 3: Fig. S3B–D) and is somewhat conical in proximal view (Fig. 8N, O). Megaspores are ca. 1.5 mm in the length of polar axis, while the body and gula are 765–1256 μm and 400–817 μm in maximum length, respectively (Fig. 8F–N; Additional file 3: Fig. S3B–D). The gula has no visible ornamentation on the surface (Fig. 8E–J, L–N; Additional file 1: Fig. S3B, C), while the body displays ornamentations of densely and evenly distributed spines (Fig. 8E–I, P–R; Additional file 3: Fig. S3E–H). The spines are often curved, ca. 85 μm in diameter at bases and taper towards apices (Fig. 8P–S; Additional file 3: Fig. S3E–H). Some parts of ornamentations could display a verrucate appearance after the spines are truncated (Fig. 8T; Additional file 3: Fig. S3I).

Comparison with *Guangdedendron micrum* described by Wang et al. [15]

Fossils in this study are collected from the same sections and morphologically consistent with *Guangdedendron micrum*, which is considered as a possible monocarpic and dioecious tree lycopsid bearing stigmarian rhizomorphs [15]. This study supports the former conclusions and adds new traits of rootlet scars, stems and axes bearing vegetative leaves, terminal megasporangiate strobili and megaspores. The length and width of rhizomorph axis are expanded, approaching in the size of *Stigmaria ficoides* from the Middle Pennsylvanian [16]. Stems of 8.0–10.0 cm width (Fig. 1A, C, E, 3A–K) and axes with dichotomy (Fig. 3L–O) are found again, but they are still unusual, thus supporting *G. micrum* as a small tree with an advanced rooting system type and a simple crown [15]. The lower portions of stems lack vegetative leaves but show leaf cushions indicating leaf abscission. Vegetative terminal twigs in Fig. 4A–C with leaves gradually shorten upwards may indicate apex of juvenile plants of *G*. *micrum*, as reconstructed in Fig. 6A in Wang et al. [15]. The strobili of *G. micrum* are large and often borne in pairs, and occasionally dichotomize once.

tituted different populations and lived far from the fossil locality, or the microsporangiate strobili are difficult to be preserved or identified.

### Comparison with Late Devonian heterosporous lycopsids bearing monosporangiate strobili in China

Despite an even larger collection of *Guangdedendron micrum*, no strobili containing microspores have been found. Previous studies of coeval heterosporous lycopsids often report mega- and microsporangiate strobili [17–19]. One possible explanation is that *G. micrum* has few male individuals and parthenogenesis happened, while other interpretations include that the male individuals consSeveral Late Devonian members of the Isoëtales *sensu lato* have been reported in China, e.g. *Sublepidodendron songziense* [17, 20, 21], *Sublepidodendron* *grabaui* [19, 22], *Minostrobus chaohuensis* [18, 23], *Changxingia longifolia* [24] and *Changxingia* sp. [25]. These taxa, together with *Guangdedendron micrum*, could be assigned to the Suborder Dichostrobiles, i.e. isoetaleans that produce monosporangiate strobili [26]. Major morphological traits of these plants are compared in Table 1, while a more detailed comparison is given (see Additional file 4: Table S1). Among these plants, most of them are recognized as arborescent, while *G. micrum* and possibly *S. songziense* bear stigmarian rooting system. *Changxingia longifolia* and *Changxingia* sp. display the smallest sizes of stems and megasporangiate strobili. *S. songziense* and *S. grabaui* possess lateral branches, but *G. micrum* bears a crown with fewer bifurcations. *G. micrum* shows the leaf bases of similar shape with *M. chaohuensis* and *S*. *grabaui*, but the latter displays only leaf bases with false leaf scars and no typical leaf cushions. Strobili of *G. micrum* are often in pairs and occasionally dichotomous, and larger than those of other coeval taxa. Alations along sporophyll pedicels are distinct in *M. chaohuensis* but relatively undeveloped in *G*. *micrum*, *C. longifolia* and *S. songziensis*. Considering the number of megaspores in each megasporangium, *G. micrum* and *S. songziense* display multiple but *C. longifolia* and *Changxingia* sp. possibly four, and *M. chaohuensis* contains four megaspores with some of them aborted. All of these plants exhibit *Lagenicula* megaspores with distinct gula, which is however larger in *G*. *micrum*.

**Table 1:** Comparisons of *Guangdedendron micrum*, *Minostrobus chaohuensis*, *Changxingia longifolia*, *Changxingia* sp., *Sublepidodendron songziense* and *Sublepidodendron grabaui*

	*G. micrum* [15]		*M. chaohuensis *[18, 23]	*C. longifolia *[24]	*Changxingia* sp. [25]	*S. songziense *[17, 20, 21]	*S. grabaui * [19, 22]
Branching system	Dichotomize at least twice		Dichotomize at least eight times	Dichotomize at least twice	–	Monocaulous trunk with dichotomized lateral branches	Dichotomize at least four times with lateral branches
Shape of leaf cushion	–	Narrow-fusiform	Narrow-fusiform	Rhomboid	Rhomboid	–	–
Shape of leaf base	Narrow-fusiform		Rhombic	Oblanceolate	Rhomboidal	Fusiform, narrow-rhomboid or oval	Narrow-fusiform
Strobili known	Megasporangiate		Mega- and microsporangiate	Megasporangiate	Mega- and microsporangiate	Mega- and microsporangiate	Mega- and microsporangiate
Attachment of terminal strobilus(i)	Singly or in pairs	Singly, in pairs or occasionally once-dichotomized	Singly	Singly	–	Singly	Singly
Megaspore number per megasporangium	–	Multiple	4, sometimes unequal in sizes	Probable 4	Over 4?	At least 20	–
Megaspore type	–	*Lagenicula*	*Lagenicula*	*Lagenicula*	*Lagenicula*	*Lagenicula*	–

–, lack of information; the data in the left and right columns of *G. micrum* are from Wang et al. [15] and this study, respectively

### Comparison with Late Devonian heterosporous lycopsids outside of South China

Several Late Devonian heterosporous lycopsids outside South China are known for their bisporangiate strobili. Two such lycopsids were recently reported from Gondwana palaeocontinent. *Cymastrobus irvingii* is a 3-D preserved bisporangiate strobilus from Famennian in New South Wales, Australia [27]. Its megasporangia contain a large number of megaspores, up to 500 μm in diameter. Casts of the megaspores show numerous circular pores surrounding the trilete mark and in several rows but no gula, thus differing from the *Lagenicula*-type megaspores in *Guangdedendron micrum*. *Kowieria alveoformis* from the Famennian of South Africa produces up to four *Lagenicula* megaspores [28] similar with those of *G*. *micrum*, while they differ in the number of megaspores per sporangium. In addition, *K. alveoformis* bears sporophylls homomorphic to vegetative leaves, in contrast with those of *G*. *micrum*. *Clevelandodendron ohioensis* is a Famennian lycopsid from the USA [29]. It displays a straight and totally unbranched stem terminated by a single bisporangiate strobilus, and contains *Triletes* megaspores and microspores. Unlike species mentioned above, *Jurinodendron* (= *Cyclostigma*) *kiltorkense* bears monosporangiate strobili and was a widespread taxon during the Upper Devonian and Early Mississippian [30]. *J*. *kiltorkense* is similar with *G. micrum* in the shape of sporophylls and *Lagenicula*-type megaspores [31]. *J. kiltorkense* differs from *G. micrum* in its small circular leaf scars (about 1.5 mm in diameter) and lacking of leaf cushions.

## Discussion

Most Devonian lycopsid fertile zones or strobili show no bifurcations [8, 32]. However, bifurcated strobili or fertile zones occurred in several taxa of the Middle-Late Devonian lycopsids, e.g. the Givetian *Yuguangia ordinata* [33], Frasnian *Kossoviella timanica* [34] and an undetermined “type C” [35], and Famennian *Hefengstrobus bifucus* [36] and *Guangdedendron micrum* (see details in Table 2). The evolutionary route of this trait is unclear. In most cases, the two resulted parts of strobili show nearly similar width as the strobilus before bifurcation. We suggest that the bifurcated strobili or fertile zones are produced by the dichotomy of an apical meristem after the shoot turns into reproductive growth. Alternatively, the last branching point on the terminal fertile axes may represent an earlier bifurcation than the transition to reproductive growth, i.e., the sequence of reproductive growth differentiation and apical meristem bifurcation controls the growth pattern of lycopsid strobili. In *G. micrum*, the strobili could be borne in pairs or bifurcated, which together indicate that the apical meristem has a relatively independent potential for bifurcation and sporophyll differentiation. Many tassel-fern species (especially *Phlegmariurus*) included in living Lycopodiales show similar dichotomized fertile zones indicating ready shift from vegetative branches to fertile branches (strobili) [37]. Large size and multi-dichotomized strobili may correspond to individual’s adaptation in this group to the epiphytic habit [37]. Strobili of *G*. *micrum* are various in growth patterns and sizes with lengths ranging from 5.0 [15] to 23.0 cm (9.6 cm on average), and individuals bearing large strobili may result in improved production of offsprings and surviving the turbulent condition near coastal area.

**Table 2:** Comparisons of lycopsids bearing dichotomized strobili

	*Guangdedendron micrum *[15]	*Yuguangia ordinata *[33]	*Hefengstrobus bifucus *[36]	*Kossoviella timanica *[28]	The undetermined type “C” [35]
Age	Famennian	Givetian	Famennian	Frasnian	Frasnian
Locality	Anhui, South China	Yunnan, South China	Xinjiang, Northwest China	Northern Russia (North Timan)	Hunan, South China
Strobili gender	Megasporangiate	Bisporangiate	Megasporangiate	Bisporangiate	–

–, lack of information

The earliest heterosporous lycopsids appeared in the Middle Devonian [33, 38, 39], and over ten heterosporous lycopsid genera have been reported so far from the Late Devonian [14]. Many of these early heterosporous lycopsids produce the gulate *Lagenicula* megaspores (see details in Table 3), while *Guangdedendron micrum* presents the relatively larger gula. *Lagenicula* appears not only in bisporangiate strobili of Givetian *Mixostrobus*, Famennian *Kowieria* as well as Carboniferous *Flemingites* [28, 38, 40, 41], but also in the megasporangiate strobili of all the members of Suborder Dichostrobiles reported from the Upper Devonian of South China [14, 25]. However, the taxa dominating the Carboniferous swamp, e.g. *Sigillariostrobus*, *Lepidocarpon* and *Achlamydocarpon*, bear megaspore types including *Tuberculatisporites* and *Cystosporites* [42–44]. We consider that there was a significant evolutionary change in the megaspore type between the early and late representatives of heterosporous lycopsids.

**Table 3:** Comparisons among some Devonian lycopsids’ fertile structures

Taxon	Gender of strobili	Spore type (microspores and megaspores, respectively)	Locality
*Yuguangia ordinata *[33]	Bi	*Acinosporites*	South China
		*Triletes*	
*Cymastrobus irvingii *[27]	Bi	*Endosporites*	Australia
		?	
*Kossoviella timanica *[34]	Bi	*Cristatisporites*	North Russia
		*Triletes*	
*Lepidostrobus xingjiangensis*[50]	M	*Lycospora*	North-west China
*Minostrobus chaohuensis *[18, 23]	M	*Lycospora*	South China
	F	*Lagenicula*	
*Sublepidodendron songziensis *[17, 21]	M	*Lycospora*	South China
	F	*Lagenicula*	
*Sublepidodendron grabaui *[19, 22]	M	*Lycospora*	South China
	F	?	
*Jurinodendron* (*Cyclostigma*) *kiltorkense *[31]	F	*Lagenicula*	Worldwide
*Kowieria alveoformis *[28]	Bi	?	South Africa
		*Lagenicula*	
*Changxingia* sp*. *[25]	M	?	South China
	F	*Lagenicula*	
*Guangdedendron micrum *[15]	F	*Lagenicula*	South China
*Longostachys latisporophyllum *[39]	F	*Laevigatisporites*?	South China
*Mixostrobilus givetensis *[38]	Bi	?	Kazakhstan
		*Lagenicula*	
*Bisporangiostrobus harrisii *[51]	Bi	*Geminospora*	America
		*Duosporites*	
*Clevelandodendron ohioensis *[29]	Bi	*Calamospora*? or *Punctatisporites*?	America
		Not *Lagenicula*	

F for female (megasporangiate), M for male (microsporangiate) and Bi for bisporangiate

During the Middle-Late Devonian, the lycopsids evolved well-differentiated strobili and sporophylls [45]. Each specialized sporophyll is composed of a pedicel and an upturned or curved lamina. Reduction in the number of megaspores per sporangium together with alations widened and upturned to enclose the sporangium are considered as evolutionary trends within the Suborder Dichostrobiles [23, 46–48]. In one megasporangium, *Minostrobus chaohuensis* contains one functional megaspore and three aborted megaspores, which is regarded as the most derived taxon among the Famennian members of Dichostrobiles [23]. As for the other taxa, *Changxingia longifolia* and *Changxingia* sp*.* have probable four megaspores per sporangium, while *Guangdedendron micrum* and *Sublepidodendron songziense* [21] show multiple megaspores that are considered primitive. On the other hand, the alations of *C. longifolia* and *S. songziensis* resemble those of *Achlamydocarpon*, which leave the sporangium largely exposed and represent a primary status, while *Minostrobus* represents the intermediate [23] and *Lepidocarpon* the most derived [46]. Although carefully serial dégagement has been applied to the *G. micrum* sporophylls in lateral and face views, the existence of alations still cannot be confirmed, but suggests undeveloped alations probably comparable with *C. longifolia* and *S. songziensis*. The relatively primitive characteristic combination of *G. micrum* hints a basal position in the lineage of Suborder Dichostrobiles. In addition, as suggested by Philips and DiMichele [49], there are many megaspores exposed on the surfaces of compressed strobili, probably indicating the free sporing habit.

## Materials and methods

Specimens of fossil plant *Guangdedendron micrum* in this study were further collected from the same localities and horizons illustrated by Wang et al. [15], i.e., the upper part (Leigutai Member) of the Upper Devonian (Famennian) Wutong Formation at Jianchuan and Yongchuan mines, Xinhang Town, Guangde City, Anhui Province, China. Several specimens observed in the previous study (including PKUB16001a, PKUB16099, PKUB16141, PKUB16144, PKUB16151 and PKUB21006, see Taxonomy subsection) are also reinvestigated. Specimens of *G. micrum* are preserved as casts, impressions and compressions. The plant morphology is exposed using steel needles. Carbonaceous fragments of the strobili with spores are either macerated with HCl and HF, or collected and fixed with conductive tape, for observation under the light microscope (LM) and scanning electron microscope (SEM), respectively. Photographs are taken with the digital camera, LM and SEM. Specimens numbered with PKUB and YC are separately housed at the Department of Geology, Peking University, Beijing, China, and Anhui Geological Museum, Hefei, China, respectively.

## Supplementary Information

**Additional file 1: Fig. S1.** Stems and strobili of *Guangdedendron micrum* from Jianchuan (A–G, I, L–O) and Yongchuan (H, K, P, Q) mines. (A, B) Two sides of a stem with expanded base and leaf cushions. PKUB21005. (C, D) Two sides of a stem. PKUB21006. (E, F) In-situ once-dichotomized stems. Two arrows in Fig. S1E indicating two daughter axes. (G) Oval fissures helically arranged along stem. (H) Oval fissures helically arranged along in-situ stem. (I) Enlargement of portion in Fig. 3R (arrow), showing a ligule pit (Lp) and a vascular bundle scar (Vs). (J) Interpretative line drawing of helically arranged leaf bases according to Fig. 3U, indicating outlines (black lines) and parastichies (red dotted lines) of leaf bases. PKUB16049. (K, L) Dichotomized fertile axes with terminal single strobilus. (M, N) Terminal strobili in pairs. PKUB16097, 16099. (O) Dichotomized fertile axis with partially preserved strobili. (P) Over ten strobili preserved in the same direction. (Q) A possibly once-dichotomized strobilus. Arrow indicating portion enlarged in Fig. 7J. PKUB16064. Scale bars: A–D (5 cm), F, L (2 cm), G, M–O, Q (1 cm), I (2 mm), J (5 mm); diameter of the coin for scale: 2 cm (E, H, K, P).

**Additional file 2: Fig. S2.** Enlargement of Fig. 5C (arrow 2), 10 stages (A–J) of serial dégagement showing structure and arrangement of sporophylls, and interpretative line drawings (A’ –J’). Scale bars = 2 mm.

**Additional file 3: Fig. S3.** SEM of megaspores of *Guangdedendron micrum* displaying body, gula and ornamentations. (A) Several megaspores. (B) Megaspore in lateral view. (C) Two megaspores. Arrows 1 and 2 indicating portions enlarged in Fig. S3E and 8 T, respectively. (D) Megaspore in lateral view. Arrow indicating portion enlarged in Fig. S3F. (E, F) Enlargement of portions in Fig. S3C (arrow 1) and S3D (arrow), respectively. Showing tapered and curved spiny ornamentations. (G, H) Tapered spiny ornamentations. (I) Persistent basal portions of spiny ornamentations with upper parts missing or abscised. Scale bars: A (500 μm), B–D (200 μm), E–H (100 μm), I (50 μm).

**Additional file 4: Table S1.**

## Data Availability

All data generated or analyzed during this study are included in this manuscript, 8 figure files, 3 table files, its Additional file 4: Table S1 and 3 additional figure files.
