# Supplementary figures and images for: Re-study of Guangdedendron micrum from the Late Devonian Xinhang forest
Source: BMC Ecol Evol. 2022 May 23;22:69. doi: 10.1186/s12862-022-02021-w (PMC9128225; doi:10.1186/s12862-022-02021-w)

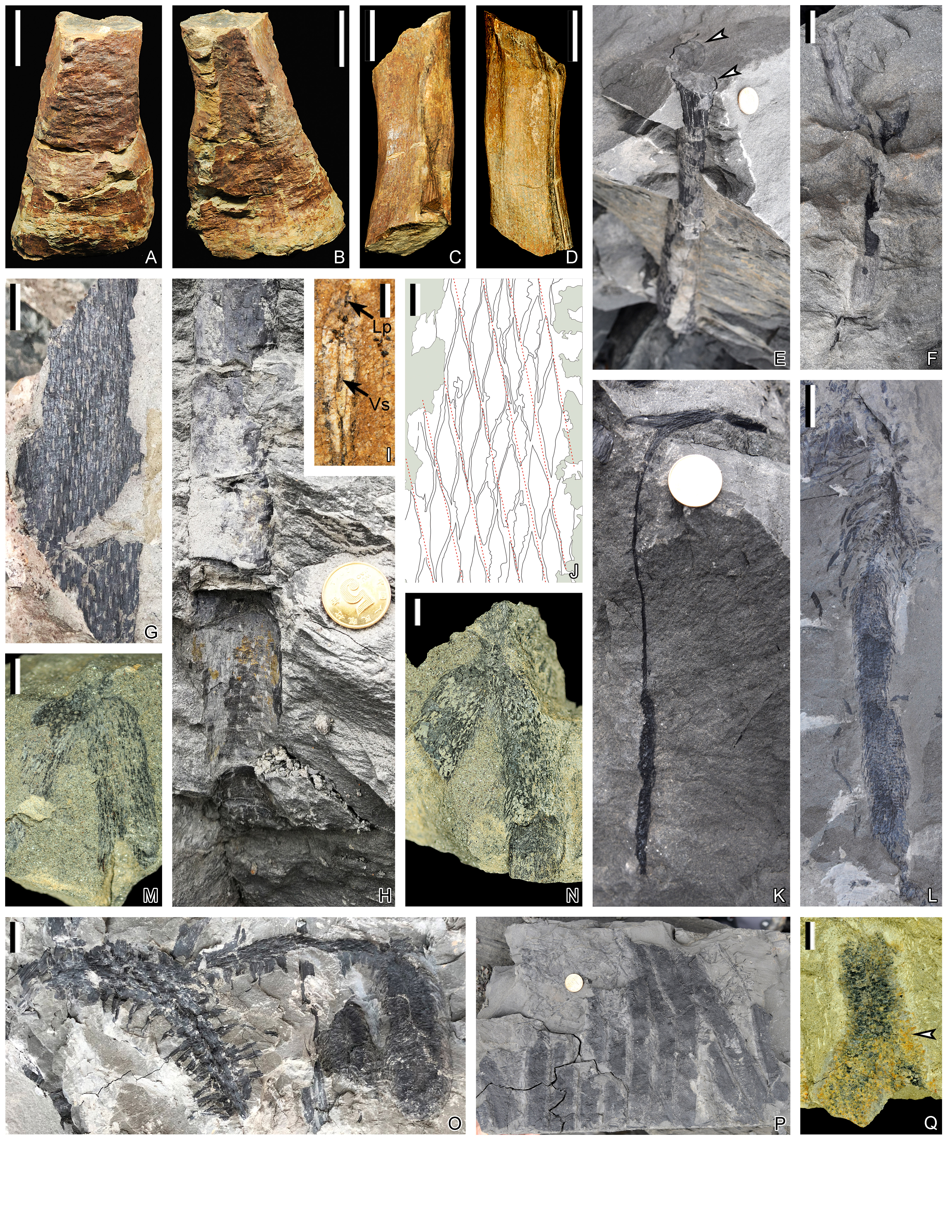

Supplement: Supplementary file 1 — Additional file 1: Fig. S1. Stems and strobili of Guangdedendron micrum from Jianchuan (A–G, I, L–O) and Yongchuan (H, K, P, Q) mines. (A, B) Two sides of a stem with expanded base and leaf cushions. PKUB21005. (C, D) Two sides of a stem. PKUB21006. (E, F) In-situ once-dichotomized stems. Two arrows in Fig. S1E indicating two daughter axes. (G) Oval fissures helically arranged along stem. (H) Oval fissures helically arranged along in-situ stem. (I) Enlargement of portion in Fig. 3R (arrow), showing a ligule pit (Lp) and a vascular bundle scar (Vs). (J) Interpretative line drawing of helically arranged leaf bases according to Fig. 3U, indicating outlines (black lines) and parastichies (red dotted lines) of leaf bases. PKUB16049. (K, L) Dichotomized fertile axes with terminal single strobilus. (M, N) Terminal strobili in pairs. PKUB16097, 16099. (O) Dichotomized fertile axis with partially preserved strobili. (P) Over ten strobili preserved in the same direction. (Q) A possibly once-dichotomized strobilus. Arrow indicating portion enlarged in Fig. 7J. PKUB16064. Scale bars: A–D (5 cm), F, L (2 cm), G, M–O, Q (1 cm), I (2 mm), J (5 mm); diameter of the coin for scale: 2 cm (E, H, K, P). [file 12862_2022_2021_MOESM1_ESM.jpg]

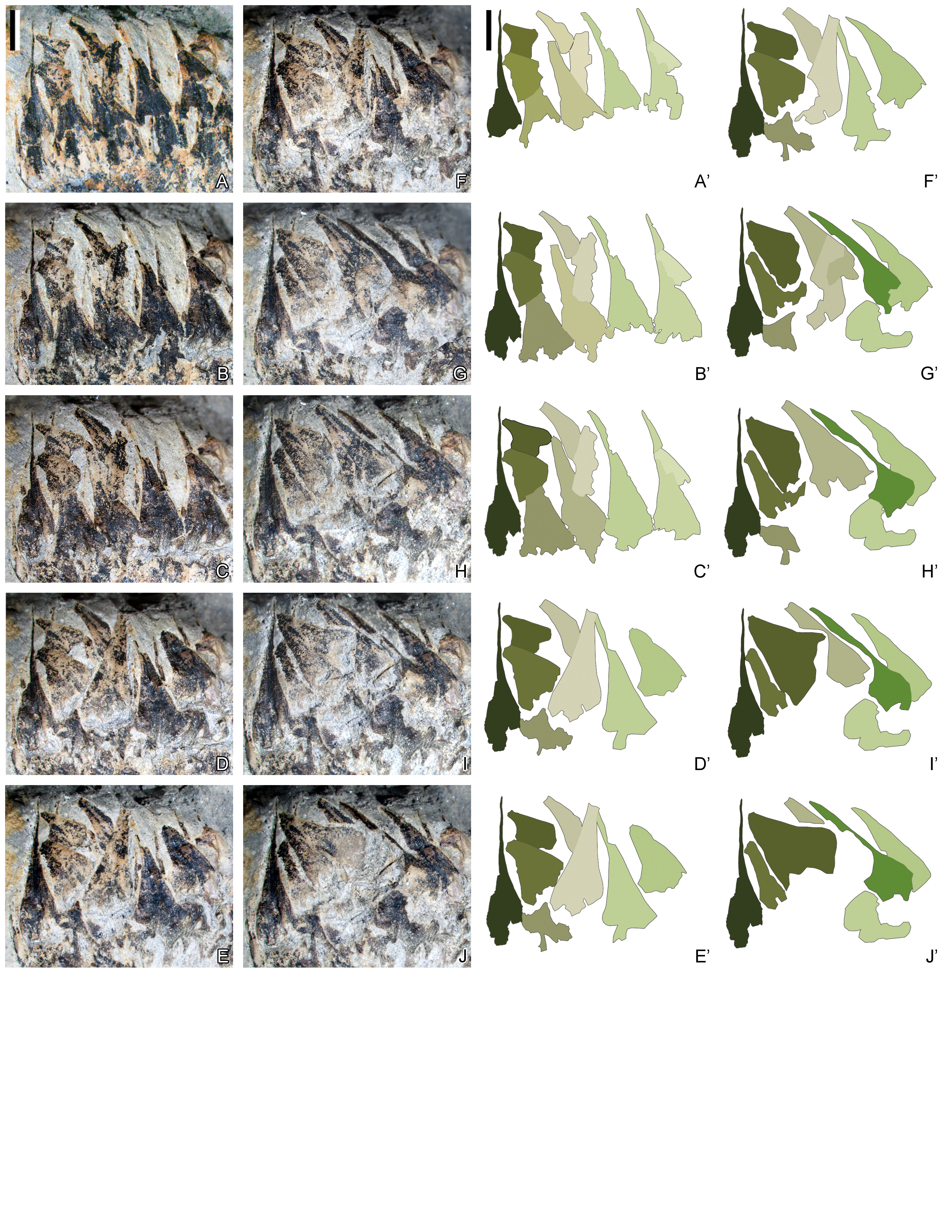

Supplement: Supplementary file 2 — Additional file 2: Fig. S2. Enlargement of Fig. 5C (arrow 2), 10 stages (A–J) of serial dégagement showing structure and arrangement of sporophylls, and interpretative line drawings (A’ –J’). Scale bars = 2 mm. [file 12862_2022_2021_MOESM2_ESM.jpg]

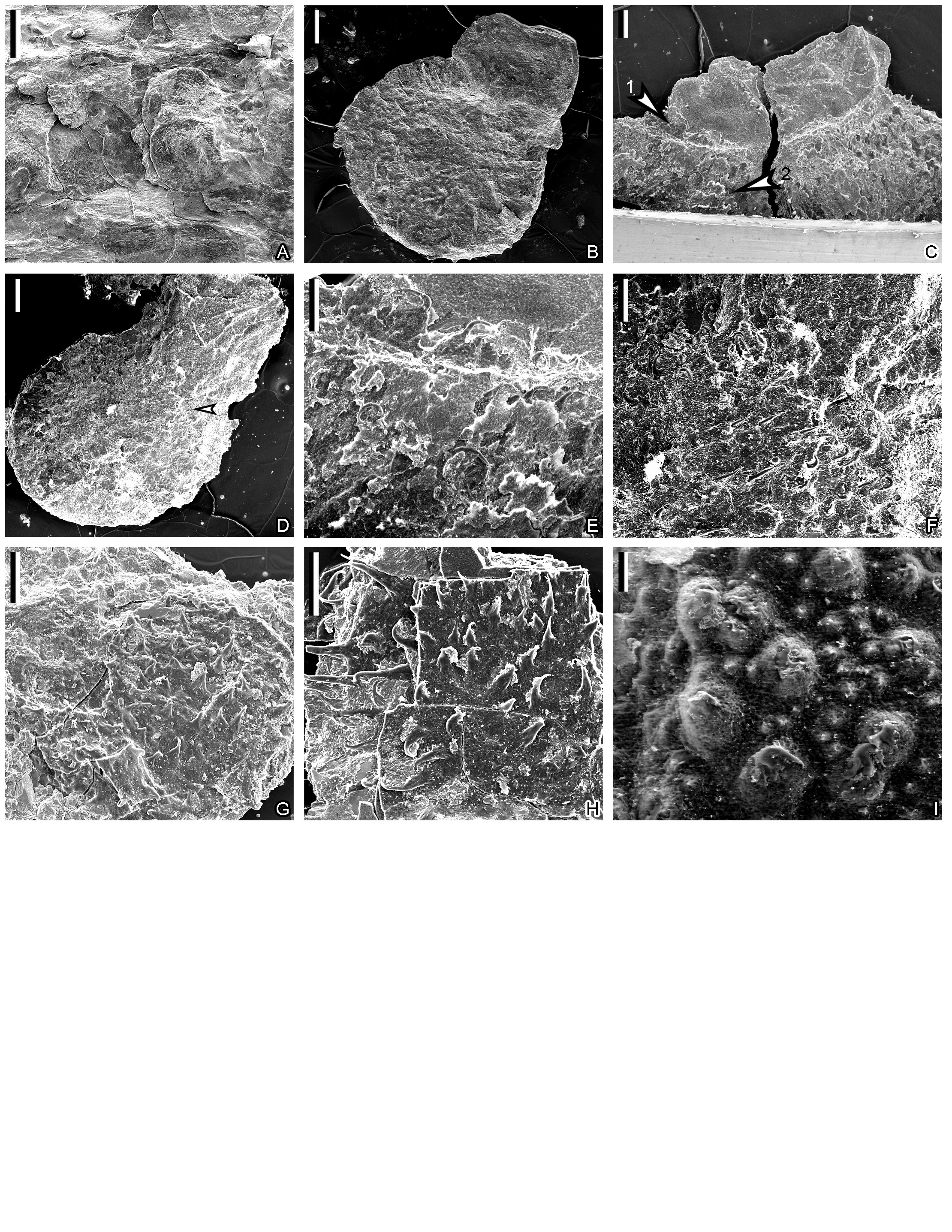

Supplement: Supplementary file 3 — Additional file 3: Fig. S3. SEM of megaspores of Guangdedendron micrum displaying body, gula and ornamentations. (A) Several megaspores. (B) Megaspore in lateral view. (C) Two megaspores. Arrows 1 and 2 indicating portions enlarged in Fig. S3E and 8 T, respectively. (D) Megaspore in lateral view. Arrow indicating portion enlarged in Fig. S3F. (E, F) Enlargement of portions in Fig. S3C (arrow 1) and S3D (arrow), respectively. Showing tapered and curved spiny ornamentations. (G, H) Tapered spiny ornamentations. (I) Persistent basal portions of spiny ornamentations with upper parts missing or abscised. Scale bars: A (500 μm), B–D (200 μm), E–H (100 μm), I (50 μm). [file 12862_2022_2021_MOESM3_ESM.jpg]
