# Supplementary material for: Re-study of Guangdedendron micrum from the Late Devonian Xinhang forest
Source: BMC Ecol Evol. 2022 May 23;22:69. doi: 10.1186/s12862-022-02021-w (PMC9128225; doi:10.1186/s12862-022-02021-w)
Supplement: Supplementary file 4 — Additional file 4: Table S1. [file 12862_2022_2021_MOESM4_ESM.docx]

Table S1. Comparisons of characters of *Guangdedendron micrum*, *Minostrobus chaohuensis*, *Changxingia longifolia*, *Changxingia* sp., *Sublepidodendron songziense* and *Sublepidodendron grabaui*.

|  | *G. micrum*^[15]^ | | *M. chaohuensis*^[18, 23]^ | *C. longifolia*^[24]^ | *Changxingia* sp. ^[25]^ | *S. songziense*^[17, 20, 21]^ | *S. grabaui*^[19, 22]^ |
| --- | --- | --- | --- | --- | --- | --- | --- |
| **Rooting system** | stigmarian | | – | – |  | stigmarian? | – |
| **Branching system** | dichotomized several times | | dichotomized repeatedly | dichotomized | – | trunk with dichotomized lateral branches | dichotomize with lateral branches |
| **Width of stem (cm)** | 0.4–18.7 | 3.9–12.2 | 2.5–5.5 | up to 2.0 | 0.1–1.2 | 5.5–7.0 | ca. 3.2–10.0 |
| **Vegetative leaf** | persistent or deciduous | | persistent on terminal axes but deciduous on stems | persistent on terminal axes but deciduous on stems | persistent or deciduous | persistent | persistent |
| Leaf shape | linear with entire margin | | lanceolate | linear with entire margin | linear? with entire margin | linear with entire margin | linear with entire margin |
| Length (cm) | 2.0–9.2 | 3.3–8.5 | 0.5–0.7 | 1.8–2.5 | – | 1.0–1.5 | 1.8–6.0 |
| Width (mm) | 1.2–4.5 | 5.3–9.0 | ca. 1.5 | 0.5–1.2 | – | 0.7–1.2 | 0.4–1.0 |
| **Leaf cushion or base** |  |  |  |  |  |  |  |
| Shape of leaf cushion | – | long-fusiform | long-fusiform | rhomboid | rhomboid | – | – |
| Shape of leaf base | long-fusiform | long-fusiform | rhombic | oblanceolate | – | fusiform, long-rhomboid or oval | elongate fusiform |
| Size (L×W, mm) | – | (10.0–25.0) × (3.0–3.7) | (6.0–9.0) × (1.0–1.6) | (10.7–12.5) × (2.0–2.3) | (6.0–8.0) × (up to 1.7) | (1.0–3.5) × (0.8–1.5) | (6.0–8.5) × (0.9–1.3) |
| Length–width ratio | – | ca. 6:1 | – | – |  | – | ca. 7:1 |
| Ligule pit | – | presence | presence | presence | presence | – | – |
| Vascular bundle scar | – | presence | presence | – | – | – | presence |
| **Fertile axis bearing strobilus(i)** | up to 6.0 cm long, 1.5–4.7 mm wide | up to 6.4 cm long, 2.1–5.5 mm wide | ca. 1.2 cm long, 1.3 mm wide | mostly short, 1.6–2.0 mm wide and reflexed distally | – | ca. 1.5 mm wide | – |
| **Strobili known** | megasporangiate | megasporangiate | mega- and microsporangiate | megasporangiate | mega- and microsporangiate | mega- and microsporangiate | mega- and microsporangiate |
| **Megasporangiate strobilus** |  |  |  |  |  |  |  |
| Attachment of terminal strobilus(i) | singly or in pairs | singly, in pairs or occasionally once-dichotomized | singly | singly | – | singly | singly |
| Size (L×W, cm) (excluding sporophyll laminae) | (5.0–21.9) × (1.0–3.0) | (up to 23.4) × (0.9–2.4) | (up to 12.5) × (0.5–0.6) | (2.0–5.0) × (0.6–1.0) | (1.0–3.5) × (0.5–0.9) | (up to 5.0) × (0.6–1.0) | (up to 16.0) × (0.8–1.0) |
| Diameter of strobilar axis (mm) | 1.2–2.0 | 1.2–3.0 | 0.9–1.3 | 0.8–1.2 | ca. 0.8 | ca. 1.0 | – |
| **Megasporophyll** |  |  |  |  |  |  |  |
| Phyllotaxy | helically arranged | | helically arranged, 2/9 helix, 35°counterclockwise | helically arranged | – | helically arranged, 6–8 sporophylls each gyre, 70°counterclockwise | helically arranged, 18 sporophylls each gyre, 30° counterclockwise |
| Length of pedicel (mm) | – | 6.0–8.0 | ca. 1.8 | 2.8–3.9 | 2.6–3.8 | ca. 3.0 | 3.0–4.0 |
| Lamina | – | long-triangular, smooth margin, 4.5–18.0 mm long, 2.4–5.8 mm at the maximum width | long-triangular, smooth margin, 6.0–7.0 mm long, 2.5–3.0 mm at the maximum width | upturned and distal part reflexed abaxially, 12.0–18.0 mm long | upturned and curved abaxially, up to 15.2 mm long, distally ca. 0.3 mm wide | smooth margin and upturned, ca. 1.0 mm long | rhomboid with pointed apex, smooth margin |
| Alation | – | possibly present | expand laterally and bend upward surrounding the sporangial base | horizontal equal to or wider than the width of megasporangium | – | expand laterally and equal to thinner than the width of megasporangium | – |
| Pedicel–lamina length ratio (by calculation) | – | 0.4–1.8 | 0.3 | 0.2 | ca. 5.0 | 3.0 | – |
| Keel | – | up to 0.5–0.8 mm high | 0.2–0.6 mm high and tapers | 1.5–2.0 mm high | – | – | – |
| Heel | – | 2.3–2.5 mm wide, 0.8–1.0 mm high | ca. 0.4 mm high | 1.7–2.3 mm high | ca. 1.7 mm high | – | – |
| **Megasporangium** |  |  |  |  |  |  |  |
| Shape | – | elongated elliptical | smooth and spherical to elliptical | ellipsoidal | elongate elliptical | elliptical | elongate elliptical |
| Size  （L×W×H, mm） | – | （4.0–7.3）× – ×（at least 1.3–1.5） | ca. 2.0 ×1.5 ×1.5 | （2.7–3.8）× – ×（1.7–1.9） | （1.5–2.8）× (0.8–1.5) ×（ca. 1.8） | 3.0 × 1.0 ×1.2 | (3.0–4.0) × – ×0.8 |
| Megaspore number per megasporangium | – | multiple | 4, sometimes unequal in sizes | probable 4 | over 4? | at least 20 | – |
| **Megaspore** |  |  |  |  |  |  |  |
| Type | – | *Lagenicula* | *Lagenicula* | *Lagenicula* | *Lagenicula* | *Lagenicula* | – |
| Shape | – | spherical body with prominent gula | pear-shape in equatorial view | spheroidal (excluding the laesura) | round to triangular in proximal or distal view | spheroidal | circular amb |
| Gula | – | distinct gula, no visible ornamentations | distinct gula | distinct gula, 300 μm long and 250 μm wide | distinct gula | hologula | – |
| Size | 670–1200 μm in diameter | ca. 1500 μm in length | 370–1490 μm in diameter | 660–910 μm long, 400–590 μm wide (excluding the laesura) | 710–830 μm long, 310–700 μm wide | 280–340 μm in diameter | 1200 μm in diameter |
| Ornamentation | – | spiny ornamentation on body and smooth on the gula | spiny ornamentation and more or less circular amb | delicate spiny | spiny? | echinate on proximal surface and densely papillate on the distal surface | – |

Note: –, lack of information.
